# Supra-complete surgery *via* dual intraoperative visualization approach (DiVA) prolongs patient survival in glioblastoma

**DOI:** 10.18632/oncotarget.8367

**Published:** 2016-03-25

**Authors:** Ilker Y. Eyüpoglu, Nirjhar Hore, Andreas Merkel, Rolf Buslei, Michael Buchfelder, Nicolai Savaskan

**Affiliations:** ^1^ Department of Neurosurgery, Translational Neurooncology Division, Medical Faculty of The Friedrich Alexander University of Erlangen-Nürnberg (FAU), Erlangen, Germany; ^2^ Department of Neuropathology, Medical Faculty of The Friedrich Alexander University of Erlangen-Nürnberg (FAU), Erlangen, Germany

**Keywords:** supra-complete tumor surgery, dual intraoperative visualization approach, supramarginal, 5-ALA, intraoperative MRI

## Abstract

Safe and complete resection represents the first step in the treatment of glioblastomas and is mandatory in increasing the effectiveness of adjuvant therapy to prolong overall survival. With gross total resection currently limited in extent to MRI contrast enhancing areas, the extent to which supra-complete resection beyond obvious contrast enhancement could have impact on overall survival remains unclear. DiVA (dual intraoperative visualization approach) redefines gross total resection as currently accepted by enabling for the first time supra-complete surgery without compromising patient safety. This approach exploits the advantages of two already accepted surgical techniques combining intraoperative MRI with integrated functional neuronavigation and 5-ALA by integrating them into a single surgical approach. We investigated whether this technique has impact on overall outcome in GBM patients. 105 patients with GBM were included. We achieved complete resection with intraoperative MRI alone according to current best-practice in glioma surgery in 75 patients. 30 patients received surgery with supra-complete resection. The control arm showed a median life expectancy of 14 months, reflecting current standards-of-care and outcome. In contrast, patients receiving supra-complete surgery displayed significant increase in median survival time to 18.5 months with overall survival time correlating directly with extent of supra-complete resection. This extension of overall survival did not come at the cost of neurological deterioration. We show for the first time that supra-complete glioma surgery leads to significant prolongation of overall survival time in GBM patients.

## INTRODUCTION

Glioblastoma is the most commonly occurring malignant brain tumor, and is counted amongst the most devastating tumor entities [1, 2]. The median survival time of 14 months remains disappointingly modest despite current multimodal treatment [3], which currently comprises three therapeutic stages. The first stage involves surgical management, with the second and third stages consisting of radiotherapy with concomitant and in most cases later with adjuvant chemotherapy with the DNA alkylating agent temozolomide [4]. The effectiveness of these subsequent therapies depends on success in achieving maximum possible resection during prior surgery. Thus, surgery exerts decisive influence on overall patient survival [5, 6].

The aim of surgery and definition of gross total or “complete” resection have been restricted to removal of the zone corresponding to contrast agent enhancement in T1-weighted MRI sequences (Tumor Zone I), as this disturbance in the integrity of the blood-brain-barrier was considered to reflect the tumor margin. Successful removal of at least 98% of this Tumor Zone I has indeed been unequivocally associated with a significantly longer survival time in comparison to lesser cytoreduction [7–9]. Nevertheless, despite removal of even 100% of this Tumor Zone I, tumor recurrences are inevitable and mostly occur at the vicinity of the resection cavity. This leads to the logical presumption, that tumor cells responsible for these recurrences must remain well beyond the tumor margins as conventionally defined by contrast agent enhancement, i.e. in the zone of perifocal edema (Tumor Zone II) [10–12]. In addition, orphan tumor cells hidden in otherwise normal in appearance brain parenchyma (Tumor Zone III) exhibit differing biological characteristics in comparison to those within the tumor core (Tumor Zone I) [13, 14].

Supramarginal tumor surgery involves resection of tissue beyond the obvious tumor mass as defined by contrast agent enhancement [15]. This currently entails blind resection of peritumoral tissue in the hope that remaining tumor cell isles invisible at a macroscopic level will also be removed, a method usually practiced in the surgical management of brain metastases [16, 17]. Further extrapolation of this approach requires the utilization of a technique leading towards a more selective and hence a more sensitive extended resection of remaining tumor cell isles encompassing all three tumor zones. DiVA meets such requirements and enables supra-complete or supra-zonal surgery. This tailored supramarginal tumor resection with maximum possible cytoreduction achieved through selective supra-zonal or supra-complete surgery beyond the main tumor bulk could therefore exert a positive influence on overall patient survival time. Practical application of this approach would require surmounting two specific difficulties. On the one hand, a selective supra-complete tumor removal approach (i.e. a tailored supramarginal resection with 100% plus) could be complicated by damage to adjacent functionally eloquent brain areas with subsequent neurological deterioration and correspondingly negative influence on overall patient survival time. On the other hand, significant shortcomings in the accuracy of intraoperative tumor visualization in zones of low tumor cell density render discrimination between pathological and healthy brain parenchyma almost impossible, which in turn results in unacceptable amounts of tumor cells left *in situ*. Currently, two technical solutions are increasingly implemented to address these problems. The first of them involves the use of intraoperative MR-imaging with integrated functional neuronavigation, which permits real-time resection control as well as three-dimensional visualization of functionally eloquent areas of the brain. The second established technique involves the use of 5-aminolevulinic acid (5-ALA, EMA approved) in surgical neuro-oncology, permitting intraoperative tumor visualization at the cellular level and thereby enabling an increase in the rate of gross total resection from 30% to 60% [18]. 5-ALA is an amino acid belonging to the group of ketocarbonic acids, representing a precursor to heme in porphyrin synthesis. The incomplete metabolism of protoporphyrin IX to heme in tumor cells leads to intracellular accumulation of the fluorescent intermediate protoporphyrin IX. Intraoperative excitation following exposure to a blue-light source permits visualization of these tumor cells. The areas of distinct fluorescence correspond to the contrast enhancing zone in MRI scans, whereas the zone of vague fluorescence lies beyond this immediately vicinity [19]. The significant optimization oftumor visualization achieved through combination of these surgical techniques enables an extended, supra-complete and tumor zone specific although safe resection than otherwise possible. These new surgical techniques result often well beyond the conventional boundaries limited to areas exhibiting contrast agent enhancement.

The clinical feasibility of DiVA has already been successfully demonstrated [20, 21]. DiVA permits for the first time practical realization of the concept of tailored supramarginal surgery through supra-complete resection without exposing the patient to heightened perioperative risk. The aim of the current study is to evaluate the extent to which this tailored supramarginal tumor removal with selective supra-zonal resection of tumor-cell isles characterized by low cell-density exerts influence on overall survival time in glioblastoma patients.

## RESULTS

### Treatment algorithm and patient distribution

**Figure 1:**
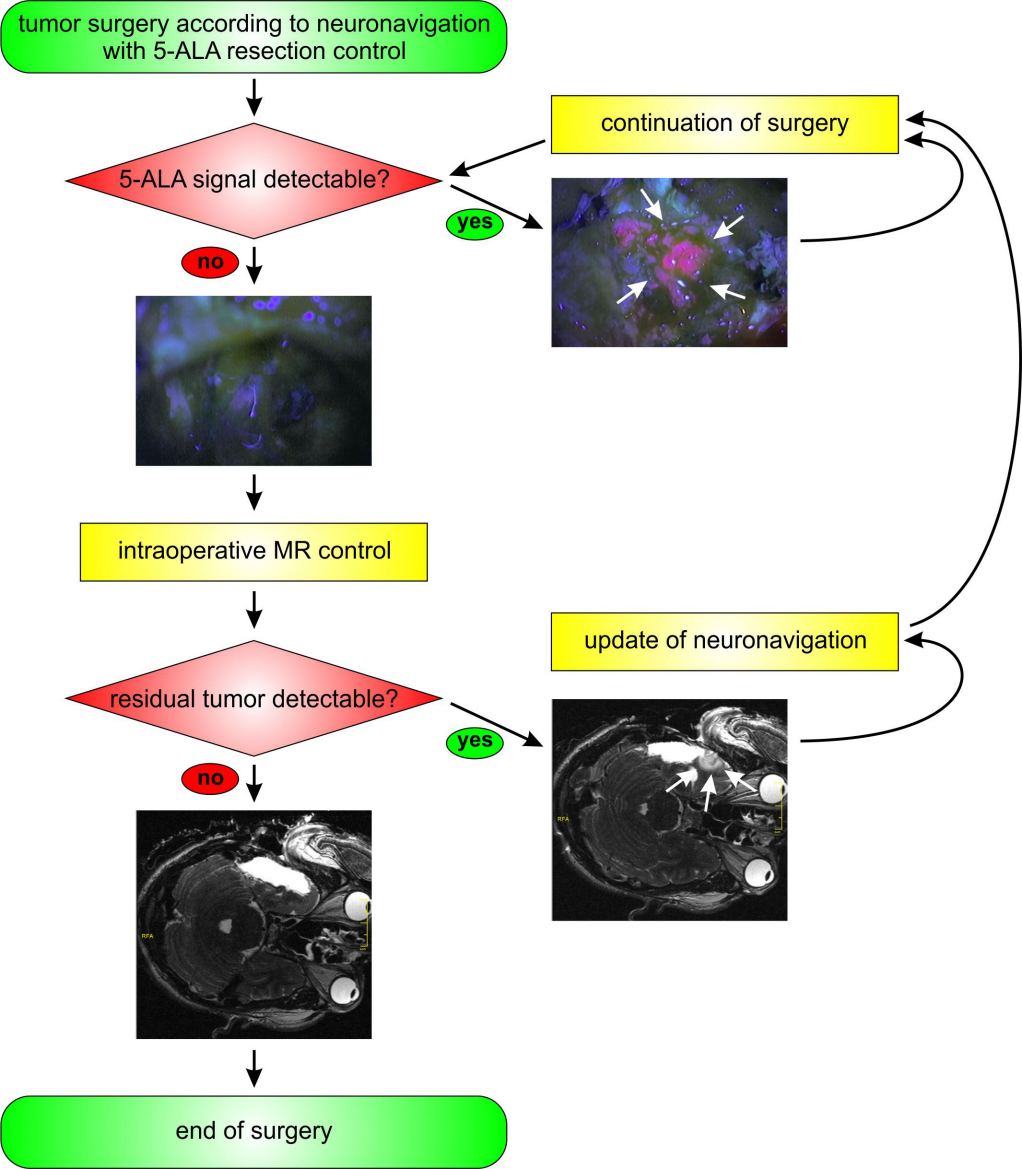
Study design and algorithm of the DiVA protocol The algorithm for surgery according to the DiVA protocol is given. Tumor resection is carried out according to functional neuronavigation and 5-ALA fluorescence until this signal is no longer detected. Following this, an intraoperative MR-scan is carried out. Should residual tumor be detected, this is then re-segmented in the neuronavigation and surgery continued according to the entire DiVA protocol. Surgery is ended at the point when the intraoperative MR-scan shows no residual tumor.

Tumor volumetry was performed immediately prior to surgery and resection then carried out according to the 5-ALA signal alone with completeness of resection defined by absence of any visible signal, i.e. both distinct and vague. This determination was carried out by the primary surgeon at all times. Functional neuronavigation data was intermittently projected to prevent inadvertent damage to functional brain areas. At the end of each stage of resection, the tumor cavity was systematically inspected to exclude residual tumor. Once the 5-ALA signal was undetectable, an intraoperative MRI scan was performed. If the extent of resection was confirmed, the decision to conclude the surgery was taken by the primary surgeon. Otherwise, the residual tumor volume was re-segmented and resection continued according to the neuronavigation. This procedure was repeated until the 5-ALA signal was no longer detectable, and the corresponding absence of contrast-enhancing tumor corroborated by iMRI (Figure 1).

**Figure 2:**
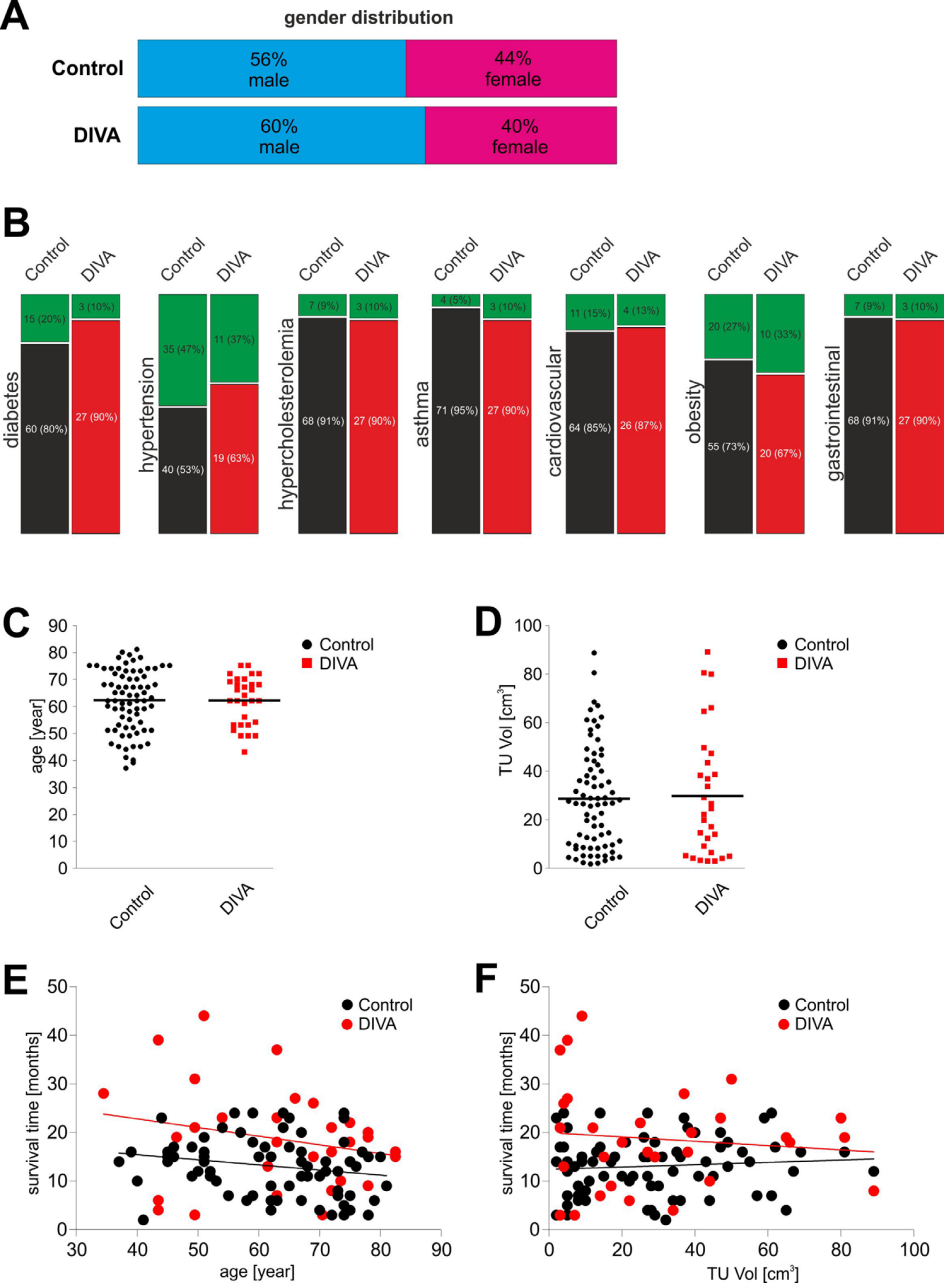
Analysis of prognostic factors (**A**) gender representation and corresponding ratios of the 105 patients were approximately equal in both groups. The control group consisted of 56% male patients (blue) and 44% female patients (red) of a total of 75 patients. In the DiVA group, 60% of patients were male (blue) and 40% were female (red) of a total of 30 patients. A one-tailed *p*-value of 0.966 in Fisher's exact test revealed an equal gender distribution in both groups. (**B**) the following comorbidities were identified: diabetes mellitus (*p* = 0.265), hypertension (*p* = 0.390), hypercholesterolemia (*p* = 1.000), bronchial asthma (*p* = 0.405), cardiovascular diseases (*p* = 1.000), obesity (defined as BMI ≥ 30; *p* = 0.486) and gastrointestinal diseases (*p* = 1.000). Selection bias was excluded by analysis through Fisher's exact test, which revealed an equal distribution in both groups. (**C**) patient age ranged from 37 to 81 years with a mean age of 62 years in the control group. Patient age in the DiVA group ranged from 43 to 75 years with a mean age of 62 years. The median age in both groups was 63 years. A *p*-value of 0.966 in *t*-test revealed no statistical difference with regard to the age distribution in both groups. (**D**) tumor volume in the control group lay between 1.8 and 88.7 cm^3^, whereas in the DiVA group between 3.0 and 89.1 cm^3^. The mean tumor volume for the control group was 28.0 cm^3^ and 30 cm^3^ for DiVA group. A *p*-value of 0.790 in *t*-test revealed no statistical difference with regard to the tumor volume distribution in both groups. (**E**) scatterplot analysis of patient age in correlation to overall survival time showed a significant decrease of overall survival time in older patients in the control group (*p* = 0.035, Spearman r), whereas no level of significance was achieved in the DiVA group (*p* = 0.306, Spearman r). Control patients are depicted in black and DiVA patients in red. (**F**) analysis by scatterplot revealed no statistically significant correlation between initial tumor volume and overall survival time in either group (control: *p* = 0.437, Spearman r; DiVA: *p* = 0.785, Spearman r). Control patients are depicted in black and DiVA patients in red.

A total of 105 patients recruited from 1st December 2001 to 28th February 2013 were included in the study with an almost equal gender representation. The control group consisted of 56% male and 44% female patients, whereas the DiVA group consisted of 60% male and 40% female patients (Figure 2A). Both study arms were characterized by even distribution of gender (*p* = 0.44, Fisher's exact test). In addition, assessed comorbidities displayed no significant difference in incidence (Fisher's exact test) (Figure 2B). Hence, comorbidities were similarly distributed in both groups (Figure 2B): diabetes mellitus (control 20%, DiVA 10%; *p* = 0.265), hypertension (control 47%, DiVA 37%;*p* = 0.390), hypercholesterolemia (control 9%, DiVA 10%; *p* = 1.000), bronchial asthma (control 5%, DiVA 10%; *p* = 0.405), cardiovascular diseases (control 15%, DiVA 13%; *p* = 1.000), obesity (control 27%, DiVA 33%; *p* = 0.486), and gastrointestinal diseases (control 9%, DiVA 10%; *p* = 1.000). The median age was 63 years in both groups with mean age of 62 ± 9 in DiVA and 62 ± 11 years in control group (Figure 2C). Age distribution was homogeneous in both study arms (*p* = 0.966, two-sided *t*-test). Distribution of tumor size was also similar in both groups with a mean volume of 28 ± 21 cm^3^ in the control arm and 30 ± 24 cm^3^ in the DiVA group (*p* = 0.932, *t*-test) (Figure 2D). Analysis of the relationship between patient age and overall survival time with a scatterplot (Figure 2E) showed a significant correlation between the two parameters in the control group (*p* = 0.035, Spearman r): the older the patient the shorter the overall survival time, whereas the DiVA group was characterized by lack of significant correlation between these parameters (*p* = 0.306, Spearman r). In addition, we found no correlation (Figure 2F) between initial tumor volume and overall survival time (Control: *p* = 0.437, Spearman r; DiVA: *p* = 0.785, Spearman r). Random testing carried out in altogether 57 (15 patients belonging to the DiVA group and 42 to the control group) of the total population of 105 patients (data not shown) showed a normal distribution with respect to MGMT promotor methylation (*p* = 0.1344, Chi-Square). Individual analysis of MGMT promotor methylation in both the control group (*p* = 0.325, Log-rank; *p* = 0.646, Gehan-Breslow-Wilcoxon) as well as the DiVA group (*p* = 0.280, Log-rank; *p* = 0.383, Gehan-Breslow-Wilcoxon) showed no significant difference in overall survival time. The distribution of tumor patients with or without MGMT promotor methylation was therefore comparable to the results of studies primarily focusing on this [22]. Both groups were thus characterized by an even distribution of major prognostic and predictive factors.

### Evolution of supramarginal surgery in to supra-zonal glioma surgery

**Figure 3:**
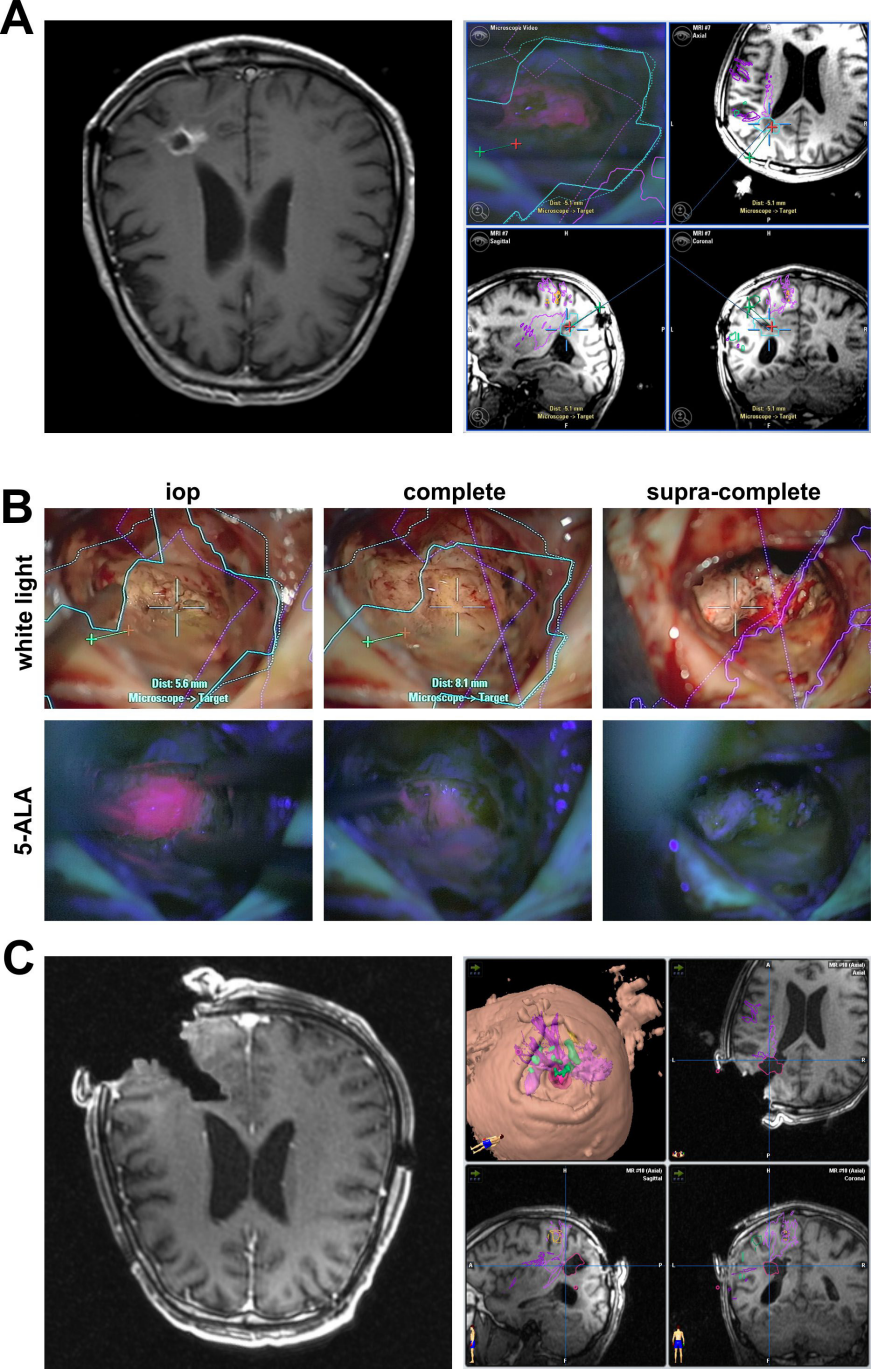
The evolution of supramarginal tumor resection in to supra-complete surgery according to the DiVA protocol (**A**) the T1-weighted MR-scans show a contrast agent enhancing space occupying lesion at the occipital horn on the left side (left image). The tumor was preoperatively segmented and the data sent to the neuronavigation (right image series). (**B**) white light microscopy is depicted in the upper row and 5-ALA fluorescence microscopy in the lower row. The left column depicts the point in surgery following resection of the primary tumor bulk, represented by a distinct 5-ALA signal. Following resection of the tumor bulk, vague 5-ALA fluorescence can be identified in the depth of the resection cavity (middle column). Conventional glioma surgery would entail ending the surgery at this point. Supramarginal resection would entail additional, unspecific peritumoral resection. In contrast, supra-complete surgery as a further refinement to conventional supramarginal resection entails selective resection of the vague 5-ALA fluorescence positive areas until no signal is detected any longer (right column). (**C**) the intraoperative T1-weighted MR-scan with contrast agent administration confirms the planned extent of resection (left image); there is no pathological contrast agent enhancement detectable any longer. Superimposition of these intraoperative images with the original segmentation demonstrates resection beyond the contrast agent enhancing areas in terms of a tailored supramarginal resection (right image series).

We first performed preoperative MR scans in both study groups with acquisition of data for neuronavigation. The contrast enhancing area in the preoperative MR images was segmented and superimposed with the neuronavigation data (Figure 3A). 75 patients underwent neuronavigation guided gross total resection strictly corresponding to the segmented area. 30 patients underwent surgery according to the DiVA protocol. The corresponding 5-ALA signal could be identified in all 30 patients with additional differentiation even between the various intensities of fluorescence (Figure 3B). The initial stage of surgery comprised of resection of the areas exhibiting the typical, distinct 5-ALA fluorescence signal, corresponding to the contrast agent enhancing areas of the MR images segmented according to the neuronavigation (Figure 3B, left column). Following this, areas of vague fluorescence signal lying outside the contrast agent enhancing areas of the MRI scans, i.e. beyond the areas segmented according to the neuronavigation was identified (Figure 3B, middle column). We performed resection of these areas until no further 5-ALA fluorescence – neither distinct nor vague – was detectable any longer (Figure 3B, right column). The subsequent intraoperative MR scans with co-referencing of the neuronavigation confirmed the extent of the tailored supramarginal (supra-complete) resection in all three tumor zones, i.e. including and beyond the MRI contrast agent enhancing areas (Figure 3C).

### DiVA is a safe surgical technique in the management of malignant gliomas

**Figure 4:**
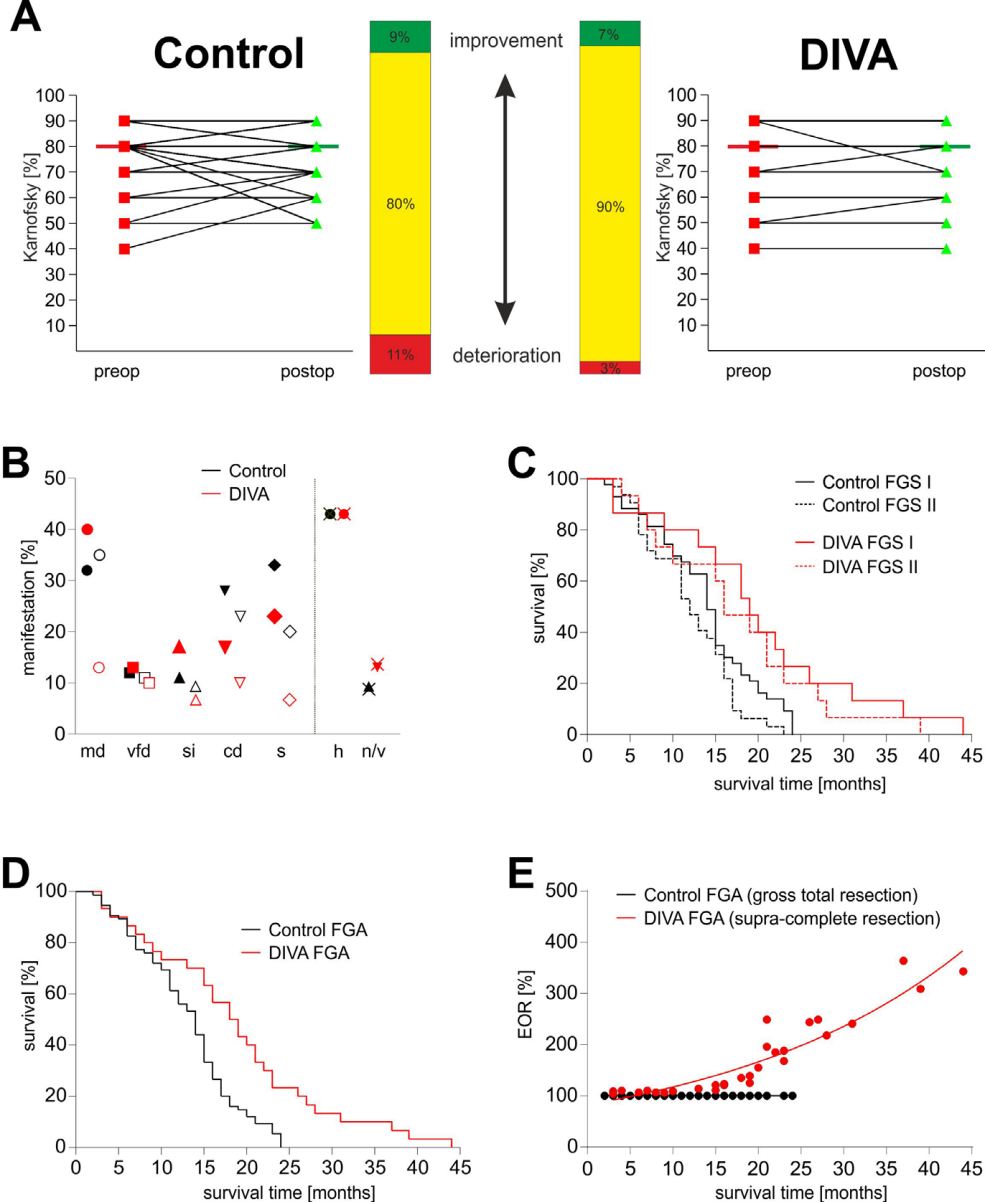
DiVA significantly prolongs overall survival in patients with malignant gliomas (**A**) the pre- (red rectangles) and postoperative (green triangles) general condition of the patients was determined according to the KPS, which remained largely unchanged between 40 to 90%. The general condition of 80% of patients in the control group and 90% of patients in the DiVA group remained perioperatively stable (yellow bars). This is reflected in the constant median KPS at 80% for both groups. With 3%, the rate of postoperative deterioration of general condition in the DiVA group was clearly lower than the 11% in the control group (red bars). 7% of patients in the DiVA group experienced postoperative improvement of general condition as opposed to 9% in the control group (green bars). A *p*-value of 0.394 for control and 0.5 for DiVA group indicates no statistical relevant deterioration in general condition in both groups (Wilcoxon matched-pairs signed rank test). (**B**) pre- and postoperative neurological status (motor deficits, visual field deficits, speech impairment, cognitive deficits, seizures) as well as non-specific symptoms (headache and nausea/vomiting) were documented. The control arm is displayed in black and the DiVA arm in red. The preoperative status is represented by a filled-in symbol and the postoperative status with an open symbol. Fisher's exact test displays no significant difference between pre- and postoperative neurological status. (**C**) Analysis of overall survival time with a classification system based on tumor localization in relation to functionally eloquent areas of the brain as determined by topography alone [24]. Analysis showed that both subgroups of patients allotted to the DiVA arm—FGS I (*p* = 0.012, Log-rank test) represented by a continuous red line and FGS II (*p* = 0.010, Log-rank test) represented by a punctuated red line—were characterized by a significantly longer overall survival time in comparison to the control arm represented by continuous and punctuated black lines respectively (Figure 4C). In addition, there was a significant difference between both general overall survival time (*p* = 0.006, Log-rank test) as well as trend analysis (*p* = 0.027, Log-rank test) in the DiVA und control arms. (**D**) overall survival time in patients with glioblastoma is depicted as a Kaplan-Meier survival curve. Control patients are depicted in black and DiVA patients in red. A *p*-value of 0.0004 was calculated in Log-rank (Chi square = 12.78) and of 0.0081 in Gehan-Breslow-Wilcoxon (Chi square = 7.008). The hazard ratio was 0.449 with a 95% CI from 0.289 to 0.696. The median survival time in the control group operated according to the current gold standard in surgical neuro-oncology was 14 months, whereas surgery according to the DiVA protocol resulted in a significantly longer median survival time of 18.5 months in the corresponding group. (**E**) correlation of extent of resection with overall survival time in patients with glioblastoma is depicted as a scatterplot. Control patients are depicted in black and DiVA patients in red. A *p*-value of 0.0001 was calculated with Spearman r, indicating a positive correlation between extent of resection and overall survival time.

The pre- and postoperative general condition of the patients was determined according to the Karnofsky performance score (KPS), which lay between 40 to 90%. The median KPS remained pre- and postoperatively constant at 80% for both groups. The general condition of 80% of patients in the control group and 90% of patients in the DiVA group remained perioperatively stable. With 3%, the rate of postoperative deterioration of general condition in the DiVA group was clearly lower than the 11% in the control group. Similarly, 7% of patients in the DiVA group experienced postoperatively improvement of general condition as opposed to the 9% in the control group (Figure 4A). The pre- and postoperative KPS was similar between the control and the DiVA group (Wilcoxon matched-pairs signed rank test, *p* = 0.394). In addition to the KPS, pre- and postoperative neurological status (motor deficits, visual field deficits, speech impairment, cognitive deficits, seizures) as well as non-specific symptoms (headache and nausea/vomiting) were taken in to account. These analyses revealed no significant difference in incidence (Fisher's exact test) and thus both groups were hence similarly distributed (Figure 4B). These findings therefore demonstrate that DiVA assisted surgery enables supra-complete resection while resulting in lower perioperative morbidity in comparison to commonly practiced techniques in glioma surgery.

### DiVA significantly prolongs the median survival time in patients with malignant gliomas

All patients underwent a uniform postoperative treatment consisting of a combined radio-chemotherapy with temozolomide. All patients were evaluated in intervals of 3 months and the time of death registered. Exclusion and inclusion criteria with respect to tumor localization were determined according to two functional grading systems: Friedlein and Sawaya. Only patients with tumors amenable to gross total resection (complete resection – FGA – functional grading A [23]) were included. Patients meeting the criteria for FGB (incomplete resection – FGB – functional grading B [23]) were excluded. For comparison, we then analyzed the same data with an older classification system based on tumor localization in relation to functionally eloquent areas of the brain as determined by topography alone [24]. Here, too, patients meeting the criteria for FGS I (functional grading system) comprising tumors located in functionally silent areas of the brain and FGS II comprising tumors located adjacent to functionally eloquent areas of the brain were included. FGS III tumors, defined as tumors located in infiltrating functionally eloquent areas of the brain, were excluded. Analysis showed that both subgroups of patients allotted to the DiVA arm—FGS I (*p* = 0.012, Log-rank test) and FGS II (*p* = 0.010, Log-rank test)—were characterized by a significantly longer overall survival time in comparison to the control arm (Figure 4C). In contrast, there were significant differences between both general overall survival time (*p* = 0.006, Log-rank test) as well as trend analysis (*p* = 0.027, Log-rank test) in the DiVA and control arms. The median survival time in the control group treated according to current gold standards in surgical neuro-oncology was 14 months, whereas surgery according to the DiVA protocol resulted in a significantly longer median survival time of 18.5 months in the corresponding group (Figure 4D). A *p*-value of 0.0004 was calculated in Log-rank (Chi square = 12.78) and of 0.0081 in Gehan-Breslow-Wilcoxon (Chi square = 7.008). The hazard ratio was 0.449 with a 95% CI from 0.289 to 0.696. The corresponding scatterplot analysis showed that extension of overall survival time was directly proportional to the extent of resection (Figure 4E): the greater the extent of resection over and above the control group (conventional gross total resection), defined here as the DiVA group (supra-complete resection), the more significant the extension of overall survival time (*p* = 0.0001, Spearman r). Patients with malignant gliomas, who underwent surgery according to the DiVA protocol, were characterized by further evolution of supramarginal surgery due to heightened precision. Therefore these patients benefited from a significant survival prolongation without attendant deterioration in general condition (Table 1).

**Table 1:** Overview of patient data and statistics

	Control	DiVA	*p*-value	Test
**no. of cases (%)**	75 (71%)	30 (29%)		
**gender**			0.440	Fisher's exact test
male	42 (56%)	18 (60%)		
female	33 (44%)	12 (40%)		
**age (year)**	→	←	0.966	Student's *t*-test
**age (year) vs survival (months)**	**0.035** ↓	0.306 ↓		Spearman r
mean ± SD	62 ± 11	62 ± 9		
median	63	63		
min - max	37 – 81	43 – 75		
**comorbidities**	→	←		Fisher's exact test
diabetes mellitus	15 (20%)/60 (80%)	3 (10%)/27 (90%)	0.265	
hypertension	35 (47%)/40 (53%)	11 (37%)/19 (63%)	0.390	
hypercholesterolemia	7 (9%)/68 (91%)	3 (10%)/27 (90%)	1.000	
asthma	4 (5%)/71 (95%)	3 (10%)/27 (90%)	0.405	
cardiovascular disease	11 (15%)/64 (85%)	4 (13%)/26 (87%)	1.000	
obesity (BMI ³ 30)	20 (27%)/55 (73%)	10 (33%)/20 (67%)	0.486	
gastrointestinal disease	7 (9%)/68 (91%)	3 (10%)/27 (90%)	1.000	
**TU Vol (cm^3^)**	→	←	0.790	Student's *t*-test
**TU Vol (cm^3^) vs survival (months)**	0.437 ↓	0.785 ↓		Spearman r
mean ± SD	28 ± 21	30 ± 24		
median	27	23		
min - max	1.8 – 88.7	3 – 89.1		
**Karnofsky**	0.394 ↓	0.5 ↓		Wilcoxon matched pairs
preop (%)				
mean ± SD	77 ± 10	77 ± 15	← 0.829	Student's *t*-test
median	80	80		
min - max	40 – 90	40 – 90		
postop (%)				
mean ± SD	76 ± 10	77 ± 14	← 0.553	Student's *t*-test
median	80	80		
min - max	50 – 90	40 – 90		
**survival (months)**	→	←	**0.0004**	Log-rank
	→	←	**0.0081**	Gehan-Breslow-Wilcoxon
mean ± SD	13 ± 6	19 ± 11		
median	14	18.5		
min - max	3 – 24	3 – 44		
**EOR (%) vs survival (months)**		↓	**0.0001**	Spearman r
mean ± SD		170 ± 75		
median		136		
min - max		104 – 364		
**survival (months) - FGS**				
FGS I	0.073 ↓	0.503 ↓		Log-rank
FGS II	0.198	0.591		Gehan-Breslow-Wilcoxon
FGS I	→	←	**0.012**	Log-rank
			0.055	Gehan-Breslow-Wilcoxon
FG II	→	←	**0.010**	Log-rank
			0.071	Gehan-Breslow-Wilcoxon
overall			**0.006**	Log-rank
trend			**0.027**	Log-rank
**preop vs. postop deficits**				Fisher's exact test
motor deficits	24 (32%)/26 (35%)	12 (40%)/4 (13%)	0.084	
visual field deficits	9 (12%)/8 (11%)	4 (13%)/3 (10%)	1.000	
speech impairment	8 (11%)/7 (9%)	5 (17%)/2 (7%)	0.648	
cognitive deficits	21 (28%)/17 (23%)	5 (17%)/3 (10%)	1.000	
seizures	25 (33%)/15 (20%)	7 (23%)/2 (7%)	0.467	

The mean, median, and smallest (min) and largest (max) numbers for both groups are mentioned. Major prognostic factors like gender, age, tumor volume and comorbidities were analyzed through the Fisher's exact test and Student's *t*-test. Scatterplots were statistically analyzed with Spearman r. The pre- and postoperative patient general condition were evaluated through the KPS and analyzed through the Wilcoxon matched pairs test. Pre-and postoperative neurological status were analyzed through Fisher's exact test. These tests did not show statistical significance, with the *p*-value set at 0.05. Overall survival time was analyzed through two independent tests, the Log-rank and Gehan-Breslow-Wilcoxon test. Both tests demonstrated a significant prolongation in overall survival in the DiVA group as opposed to the control. Overall survival time according to functional grading system according to Sawaya (FGS) was additionally analyzed through Fisher's exact test, which demonstrated a significant prolongation of overall survival in Log-rank in the DiVA group as opposed to the control group. Significant data were marked in bold and circled.

## DISCUSSION

The DiVA protocol permits selective removal of glioma-infiltrated tissue in all three tumor zones. A cardinal axis for practical execution is based on biochemical visualization through the 5-ALA signal, with differentiation between a distinct and vague fluorescence. Usually, only tissue exhibiting a distinct fluorescence signal is resected, corresponding to the pathological MRI contrast agent enhancement [25, 26]. Further resection extending to tissue exhibiting a vague fluorescence is rarely carried out despite confirmed presence of histologically significant tumor cell densities [19, 27]. This stems from the fear that extensive resection could dramatically increase the risk of damage to functionally eloquent brain areas with attendant neurological deterioration. It is here that another cardinal axis of the DiVA protocol comes in to play: the concomitant implementation of intraoperative MRI with integrated functional neuronavigation permits real-time visualization of functionally eloquent brain areas and therefore the possibility of precise control over the extent of feasible resection [28, 29]. Our current study effectively demonstrates that the natural synergy between the two techniques now permits active realization of the concept of tailored supramarginal tumor surgery without incurring neurological deterioration. What is important is that the execution of any form of selective supra-complete glioma surgery mandates the integration of fluorescence-guided surgery with any concomitant form of intraoperative functional data, e.g. electrophysiological mapping or awake-surgery as alternatives to intraoperative MRI [30–33].

Integration of intraoperative MRI with 5-ALA lends the advantage of precise spatial tumor visualization with a resolution approaching the cellular level in relation to functionally eloquent areas. This can only partially be achieved in the case of electrophysiological mapping and not at all with the 5-ALA approach alone. Correspondingly, DiVA is logically associated with a significantly reduced risk of potential damage to eloquent areas in comparison to conventional surgical approaches. The fact that gross total resection is characterized by significant prolongation of overall survival time as opposed to subtotal resection has been clearly demonstrated in a multitude of current studies [18, 34–36]. The quintessence here boils down to the simple fact that the lesser the residual tumor volume, the longer the overall survival time [36]. The extent to which a potentially dangerous supramarginal resection may influence overall survival time in glioma patients, has to date not been comprehensively investigated [37, 38]. Despite the significant advantage of large numbers of cases included [38], prior studies were limited by the fact that both arms of the studies were retrospective: this lead to the erroneous assumption that incidental resection of the T2-weighted Flair signal alterations—prone as they are to indiscriminate sensitivity to neuronal change of any kind —beyond the regular T1-weighted contrast enhancing areas was synonymous with supra-complete resection as opposed to the non-target specific supramarginal resection it probably corresponded to. Nevertheless, even these completely retrospective studies demonstrated clear prolongation of overall survival time following resection beyond the regular T1-weighted contrast enhancing areas. As a refinement to the conventional supramarginal approach, the DiVA protocol entails a supra-complete, far safer removal of tumor cell isles due to the selective nature of guided resection under direct visualization. The significance of this study lies in the clear demonstration that glioma surgery according to the DiVA protocol as opposed to current standards in neurosurgical management leads to a significant prolongation in median survival time in glioblastoma patients of 4.5 months. These data provoke the question how such small remaining tumor cell isles exert such significant influence on overall survival time.

In order to arrive at an understanding, it is necessary to delve into the treatment algorithm for malignant gliomas: following primary surgical cytoreduction, remaining tumor cell populations are further decimated by subsequent adjuvant radio-chemotherapy. The naturally aggressive cell populations surviving treatment are characterized by heightened and increasing resistance either from the very beginning or as a paradoxical development through de novo genetic mutation, laying open the possibility of iatrogenically induced negative selection. This working hypothesis is supported by the observation that the interval to recurrence in glioblastoma patients shortens with every treatment cycle [13]. Further support for this observation stems from the fact that glioblastoma recurrences almost always occur in the immediate vicinity of the initial tumor resection cavity [39]. Surgery according to the DiVA protocol enables extensive but selective resection of such tumor cell isles, as a result of which the number of remaining tumor cells to be potentially subjected to future negative selection is greatly reduced to probably only consist of solitary orphan tumor cells not detectable according to current intraoperative visualization approaches. Since the number of residual tumor cells is critical for the development of chemo-resistance, supramarginal DiVA surgery thus leads to significant prolongation of overall survival time by enabling a superior response to adjuvant therapy. The relation between tumor load and responsiveness towards chemotherapy is supported by earlier observations on low grade gliomas, where it was shown that even partial resection lead to significant prolongation of overall survival time in comparison to conservative treatment options [40]. The postulated explanation for this was that tumor debulking lead to prolongation of the time to secondary malignization. Although we are dealing with an already highly malignant tumor entity in the case of glioblastoma, further malignization characterized by heightened resistance to chemotherapy is nevertheless possible. A currently known example would be resistance to chemotherapy in glioblastoma cells exhibiting MGMT methylation. Additional mechanisms certainly exist, the identification of the underlying biology of which poses significant challenges to molecular neuropathology.

Although implementation of the DiVA protocol in glioblastoma patients lead to a significant prolongation of median overall survival time, critical evaluation of our study shows that not all patients profited equally. This is reflected in the minimum survival time of 3 months in both groups. The current lack of a means to pre-select patients who might profit from surgery according to the DiVA protocol will have to be managed in future through the establishment of clear criteria, which will require further analysis of the individual glioblastoma subgroups through randomized prospective studies.

Another weakness lies in the inability to completely exclude bias due to the single center nature of this study. The aim of such a recent study combining low-field iMRI and 5-ALA in the surgical management of glioblastoma patients lay in evaluating the extent of resection and came to the expected conclusion that this combination resulted in more frequent gross total resection, which unsurprisingly lead to a longer overall survival time than in patients undergoing subtotal resection [41]. There was neither mention of the use of functional neuronavigation nor was the issue of supramarginal resection tackled, as a result of which the extent to which the latter exerts an influence on overall survival time remains unclear. Our data on the other hand clearly represents the first step towards ethical justification for the standardization of tailored supramarginal resection in the neurosurgical arsenal against malignant gliomas by setting a critical foundation for mandatory future multicenter studies. The prolongation of median overall survival time from 14 to 18.5 months achieved in our study represents a long overdue, groundbreaking advance in the treatment of glioblastoma patients.

A further point of contention lies in the mixture of retrospective analysis of the control arm undergoing surgery according to intraoperative MRI alone and prospective analysis of the group operated according to the DiVA protocol in this study. Many prospective studies based on conventional gold standards in neurooncological treatment strategies for glioblastomas—which have remained unchanged over the years and remain current to date—have failed to report a median overall survival time beyond 14 months, something which continues to represent the toughest evaluation criterion [28, 41]. Our retrospective control arm is no different in this respect, reflecting the same 14 month median overall survival time achieved through the very same unchanged gold standards in neuro-oncological treatment strategies. Since a prospective randomization of a surgical control group would not uncover any novel aspects per se in the treatment of glioblastoma patients, the retrospective nature of our control arm consequently retains equivalent validity. Although the results arrived at through retrospective analysis of our control arm concur with overall survival times for the standard neuro-oncological management of malignant gliomas as reported in the current literature [18, 25, 41], future randomized multicenter trials will have to be conducted to minimize single center bias and consolidate our results through a bigger cohort.

Since the superiority of the DiVA protocol over conventional surgical approaches has been demonstrated in our present study, it can readily serve as a foundation for the identification and prospective analysis of the DiVA subgroups in future trials. One such focus ought to revolve around the identification of future DiVA non-responder patients and close examination of the relationship between possibly insufficient resection of tumor cell isles exhibiting vague 5-ALA fluorescence and overall survival time. Perhaps controlled amplification and quantification of increasingly vague 5-ALA fluorescence intensities to weed out areas of correspondingly lower tumor cell densities could be a potential angle of future investigation. Since solitary orphan tumor cells remaining in Tumor Zone III despite supra-complete surgery lead to tumor recurrences [14], improved intraoperative visualization to enable specific targeting of these tumor cell subpopulations continues to represent a challenge to surgical neuro-oncology.

## MATERIALS AND METHODS

### Study design and ethics

This is a parallel-group single-center trial (non-AMG, non-MPG) conducted in the Department of Neurosurgery of the Medical Faculty of the University of Erlangen-Nürnberg. The use of intraoperative MRI in DiVA was approved by the local Ethical Committee of the University. Written informed consent was obtained from all participants involved in the study. The study complies with the current laws of the Federal Republic of Germany.

### Histopathology and definition of MGMT status

The histopathology was performed by an experienced neuropathologist (R.B.) and tissue samples classified according to the current WHO classification of tumors of the CNS. For defining the MGMT methylation status five CpG sites in the MGMT promoter were analyzed using the PyroMark MD System (Qiagen). Subsequent sample preparation and pyrosequencing was performed as described in the Pyro-Mark MD Sample Prep Guidelines using the PyroMark Q24 CpG MGMT kit (part number 970032 Qiagen).

### Distribution of patients included in the study

A total of 105 patients were included in the study. All patients were operated with the assistance of intraoperative MRI with integrated functional neuronavigation. Inclusion criteria were gross total resection with complete resection of contrast enhancing areas confirmed by intraoperative MRI and definitive neuropathological assessment as primary glioblastoma (WHO °IV). The exclusion criteria was not meeting any one of the inclusion criteria, mandatory anticoagulation with thrombocyte aggregation inhibitors, permanent ferromagnetic implants, body weight higher than permissible for the iMRI operating table, pregnancy and lactation. Patients for which a lobectomy was anticipated were automatically excluded from the study. None of the study patients underwent implantation of carmustin wafers.

Retrospective data from 75 patients operated between 1st December 2001 and 30th November 2010 were included in the control group, of which 42 were male patients and 33 were female. The aim of surgery was considered to have been achieved when exclusively the contrast enhancing areas in the T1-weighted MPRAGE sequences was completely resected.

Prospective data from 30 patients operated between 1st February 2009 and 28th February 2013 were included in the DiVA group, of which 18 were male patients and 12 female. The aim of surgery was considered to have been achieved when conclusive resection beyond the contrast enhancing areas in the T1-weighted MPRAGE sequences was carried out.

### Technical approach of DiVA

The DiVA patients received an oral dose of 20 mg/kg bodyweight of a freshly prepared solution of 5-aminolevulinic acid by dissolving 1.5 g of 5-aminolevulinic acid in 50 ml drinking water 3 h before induction of anesthesia according to previously published protocols [18].

Primary surgery was fluorescence guided (for DiVA patients) with subsequent evaluation with a Siemens Magnetom 1.5 Tesla intraoperative MRI scanner with integrated BrainLab VectorVision neuronavigation.

A Carl Zeiss OPMI Pentero operating microscope with Xenon white light as well as a blue light source for fluorescence imaging was used with co-registration in the BrainLab Vector Vision neuronavigation system. MRI sequences utilized were T1-weighted MPRAGE with contrast, T2-weighted, and Diffusion-weighted. Additionally, BOLD functional MRI studies as well as Diffusion Tensor Imaging sequences were integrated. Tumor volumetry was carried out with the iPlan Cranial 3.0 software (Brainlab, Feldkirchen, Germany) according to the T1-weighted MPRAGE 3D data sets through pre- and intraoperative segmentation.

The Karnofsky Performance Scale Index (KPS) was the primary clinical assessment factor.

### Dual intraoperative visualization approach (DiVA) protocol

DiVA surgery was performed according to the previously published protocol [20]. The additionally resected tissue detected by the intraoperative MRI was also analyzed by an experienced neuropathologist, confirming pathological glioma cell infiltration.

### Statistical methods

Statistical significance was calculated with GraphPad Prism v5.02 (GraphPad Software, La Jolla, CA, USA). A *p*-value ≤ 0.05 was considered statistically significant. The Log-rank and Gehan-Breslow-Wilcoxon tests were used for statistical analysis of survival data. Hazard ratio and its adjusted 95% confidence interval were calculated. Survival of patients was shown in a Kaplan-Meier-diagraph. The student's *t*-test (*t*-test) was used for statistical analysis of age and tumor volume distributions. Gender distribution was analyzed by Fisher's exact test. Differences in pre- and postoperative general condition were analyzed by Wilcoxon matched-pairs signed rank test. Scatterplots were analyzed by Spearman *r* test.
